# Empowerment of Newly Graduated Nurses in Six European Countries 1 Year Post-Graduation: Cross-Sectional Study and Psychometric Evaluation

**DOI:** 10.1177/10436596251414412

**Published:** 2026-01-27

**Authors:** Renáta Zeleníková, Lenka Štureková, Petr Bujok, Helena Leino-Kilpi, Liisa Kuokkanen, Alvisa Palese, Laura Visiers-Jiménez, Anna Brugnolli, Jana Nemcová, Célia Simão De Oliveira, Marília Rua, Satu Kajander-Unkuri

**Affiliations:** 1 University of Ostrava, Czech Republic; 2 Palacký University in Olomouc, Czech Republic; 3 University of Turku, Finland; 4 Wellbeing Services County of Southwest Finland, Turku University Hospital, Finland; 5 University of Udine, Italy; 6 San Juan de Dios Foundation, Madrid, Spain;; 7 Comillas Pontifical University, Madrid, Spain; 8 Azienda Provinciale per i Servizi Sanitari, Trento, Italy; 9 Comenius University in Bratislava, Slovakia; 10 Lisbon School of Nursing, Portugal; 11 University of Aveiro, Portugal; 12 Tampere University, Finland

**Keywords:** culturally adaptable, empowerment, Europe, newly graduated nurse, reliability, psychometrics, validity

## Abstract

**Introduction:**

Global nursing shortage emphasizes empowering newly graduated nurses (NGNs) to enhance retention and satisfaction. While empowerment is widely studied, little is known about NGNs’ empowerment 1 year post-graduation. This study describes it and evaluates the Essential Elements of Nurse Empowerment (EENE ©Kuokkanen 2003) instrument.

**Methods:**

In total, 239 NGNs from six European countries responded to this cross-sectional study using the EENE in 2019–2020. Data were analyzed statistically.

**Results:**

Most NGNs reported moderate empowerment. NGNs planning to stay in their workplace and profession reported higher empowerment. Exploratory factor analysis supported a four-factor structure, and the Cronbach’s alpha for the instrument was 0.88.

**Discussion:**

Empowering NGNs is crucial for improving job satisfaction and retention. Inclusive practices and validated, culturally adaptable instruments like the EENE support equitable workforce development and culturally adaptable care. “Culturally adaptable” means retaining conceptual meaning across languages and contexts; translation revealed cultural nuances in how empowerment is understood and expressed.

## Introduction

The shortage of nurses continues to present a significant challenge to health systems worldwide, undermining the capacity to deliver equitable, safe, and high-quality care across diverse populations (Tamata & Mohammadnezhad, 2023). Within this context, nurse empowerment has gained recognition as a critical factor in enhancing job satisfaction and retention and in improving patient outcomes and organizational performance (Yesilbas & Kantek, 2024).

Although widely employed in nursing discourse, the concept of empowerment lacks a universally accepted definition. It is often described as both a process and an outcome, encompassing personal growth and professional development (Goedhart et al., 2017). The theoretical foundation of empowerment in nursing comprises four main perspectives: organizational/structural theory, psychological theory, critical social theory (Kuokkanen & Leino-Kilpi, 2000), and mixed theory, which integrates elements from all preceding frameworks (Kennedy et al., 2015). Structural empowerment plays a pivotal role in fostering a positive workplace culture (Fragkos et al., 2020), emphasizing factors such as nurses’ perceptions of empowerment and leadership, alongside work-related outcomes including engagement, job satisfaction, organizational commitment, and intention to leave (Fragkos et al., 2020; Woodward, 2020). Psychological theory, on the contrary, conceptualizes empowerment as intrinsic task motivation, comprising four key dimensions: competence, impact, meaning, and self-determination. This perspective highlights the internal processes that confer a sense of control and fulfillment in one’s work (Friend & Sieloff, 2018; Spreitzer, 1995). Critical social theory is often associated with improving the quality of life for marginalized groups and advocating for social justice. It aims to challenge and transform oppressive social structures and power imbalances, thereby promoting empowerment at a broader societal level (Kennedy et al., 2015; Kuokkanen & Leino-Kilpi, 2000).

Empirical studies have shown that empowered nurses are more likely to report higher levels of job satisfaction (Gu et al., 2022; Hjazeen et al., 2024), reduced turnover intentions (Favaro et al., 2021), stronger collaboration with colleagues, and increased willingness to pursue leadership and advanced practice roles (Ta’an et al., 2020). Furthermore, empowerment has been linked to the development of a healthy work environment, which is essential for retaining nursing staff and ensuring patient safety (Dirik & Seren Intepeler, 2024; Goedhart et al., 2017).

While available literature has concentrated on empowerment among experienced nurses (e.g., Kuokkanen et al., 2014; Ta’an et al., 2020), comparatively less attention has been devoted to newly graduated nurses (hereafter NGNs), particularly 1 year post-graduation—a period widely recognized as critical for retention, career trajectory, and overall well-being (Gong et al., 2022; Zhang et al., 2025). NGNs often face transition shock when shifting from academia to clinical practice, leading to stress, dissatisfaction, and early turnover (Xin et al., 2024; Zhang et al., 2025). Empowered nurses report higher satisfaction and are more likely to stay (Gu et al., 2022) ensuring care continuity and mitigating shortages (de Vries et al., 2023; Şenol Çelik et al., 2024). However, the experience and impact of transition shock may differ across cultural and health care system contexts, reflecting variations in role expectations, organizational support, and culturally embedded communication norms (Yingnan et al., 2024).

Empowerment is not a culturally neutral construct; rather, it manifests differently across diverse cultural contexts. Within transcultural nursing, empowerment enhances nurses’ confidence, autonomy, and professional competence, thereby enabling the provision of culturally safe and responsive care (Presley & Mokoboto-Zwane, 2023). Empowered nurses are more likely to engage in reflective practice and to advocate effectively for patients from diverse backgrounds, which contributes to improved quality and equity of care. Moreover, empowerment extends beyond an individual or professional attribute—it represents a key mechanism for fostering culturally competent health care systems, promoting nurse retention in multicultural workplaces, and advancing culturally congruent leadership (Jacobson et al., 2005).

Several studies have indicated that the final year of nursing education affects graduates’ career intentions (e.g., Anyango et al., 2024; Kaihlanen et al., 2021); however, the first-year post-graduation may be even more decisive in determining whether a nurse remains in the profession (Gong et al., 2022; Zhang et al., 2025). Supporting NGNs’ empowerment during this formative phase contributes to the development of a robust professional identity, enhances self-confidence, and promotes long-term retention (Baharum et al., 2023; Kuokkanen et al., 2016). Therefore, our study builds upon the Ideal Model of Empowerment developed by Kuokkanen and Leino-Kilpi (2001), which conceptualizes work-related empowerment as a process of professional growth and development. The model forms the basis of the Essential Elements of Nurse Empowerment (EENE) instrument, which comprises four elements: “Qualities,” “Performance,” “Promote,” and “Impede” (©Kuokkanen, 2003), described as follows: (a) “Qualities” refers to a “competent nurse who has personal power and is autonomous and personally responsible,” and who is “innovative, enthusiastic, a promoter, and forward-thinking”; (b) “Performance” denotes a nurse who “discusses openly, works toward a common goal, and solves problems”; (c) “Promote” encompasses factors that facilitate work empowerment; and (d) “Impede” includes factors that hinder work empowerment (Kuokkanen, 2003, p. 42). The instrument has previously been employed in a large, cross-national study involving graduating nursing students (Visiers-Jiménez et al., 2022), but has not yet been tested among nurses who have completed their first year of clinical practice.

The aim of this study is twofold: (a) to describe the level of empowerment of NGNs 1 year post-graduation in six European countries and (b) to evaluate the psychometric properties of the EENE instrument in an early-career, international cohort of NGNs.

## Method

### Design and Setting

This cross-sectional study forms part of a longitudinal project COMPEUnurse (Competence of Nursing Students in Europe) involving nurses from the Czech Republic, Finland, Italy, Portugal, Slovakia, and Spain at the point of graduation (https://sites.utu.fi/nursingscienceresearchprogrammes/pedagogic/projects). The present study focuses on the empowerment of NGNs following their first year in the nursing profession, and findings are here reported in accordance to the STROBE checklist (von Elm et al., 2014).

### Sample

Convenience sampling was employed. Nurses were initially included at the time of graduation based on the following criteria: enrollment in a nursing degree program leading to qualification as a Registered Nurse, and being in the final stage of the program, approaching graduation (Visiers-Jiménez et al., 2022). Upon completing the initial survey, participants were invited to provide their contact details for the purpose of a follow-up survey 1 year later. These details were stored in a separate file by the principal investigator to ensure they could not be linked to individual survey responses. One year later, the principal investigator contacted those participants (*n* = 1,742) who had voluntarily provided their contact information; of these, 239 completed the follow-up questionnaire.

### Measures

#### Background Factors

Those potentially associated with the level of empowerment included age, gender, intention to leave the nursing profession, intention to change the current workplace, change of workplace within the past 12 months, satisfaction with orientation at the current workplace, satisfaction with the current job, with the quality of care at the current workplace, and with nursing as a profession. Given the limited research on NGNs’ empowerment, these variables were primarily derived from those examined in a related study focusing on nurses’ competence (Koskinen et al., 2023).

#### The EENE

NGNs’ level of empowerment was assessed using the EENE (©Kuokkanen, 2003) instrument based on the original 74-item Ideal Model of Empowerment and comprising four elements: The Qualities element includes two categories: Expertise (six items) and Future Orientedness (three items); Performance includes the category Sociability (three items); Promote includes Expertise (four items); and Impede includes Personal Integrity (three items). Although two categories share the name Expertise, the items within each are distinct (Kuokkanen, 2003). This study marks the first application of the EENE instrument among NGNs since its development. The three categories of Qualities and Performance have previously been used only in a study involving European graduating nursing students, where Cronbach’s alpha values ranged from 0.69 to 0.86 across the three categories, with an overall reliability score of 0.90 (Visiers-Jiménez et al., 2022).

Items on the EENE are rated using a Visual Analogue Scale (VAS) ranging from 0 (*does not apply to me at all*) to 100 (*fully applies to me*). Empowerment levels are categorized as low (VAS 0–50), moderate (>50–75), and high (>75–100). Permission to use and translate the instrument was obtained from the copyright holder prior to the study. Following consent, a back-translation process was conducted into the required languages, and the instrument was subsequently piloted (Visiers-Jiménez et al., 2022). Although the EENE was designed to be culturally neutral and translatable, minor adaptations were needed to ensure conceptual equivalence across countries. Phrases were adjusted to fit local terminology, health care systems, and communication styles, especially regarding professional roles. All changes were made in collaboration with local experts and bilingual reviewers to preserve item intent.

### Data Collection

Data were collected via an electronic survey between February 2019 and November 2020. The timing of data collection was scheduled to occur approximately 1 year after each participant’s original graduation month and year. The principal investigator distributed the survey to those (*n* = 1,742) who had provided their email addresses during the initial data collection phase. To enhance the response rate, two reminder emails were sent.

### Data Analysis

Continuous variables are presented as means and standard deviations (SDs). Missing values were not included in the data analysis. Means were calculated only for participants who responded. Pearson’s and Spearman’s correlation coefficients were used to test associations between variables. To test differences between groups, the Mann–Whitney, Kruskal–Wallis, and Chi-square tests were used. To assess potential attrition bias, we compared background variables between the 239 nurses who responded in this study and the original cohort of 1,742 respondents. To assess the psychometric properties of the instrument, both validity and reliability were evaluated. The Kaiser–Meyer–Olkin measure of sampling adequacy and Bartlett’s test of sphericity were performed, followed by exploratory factor analysis (EFA) with cluster (oblique) rotation. Cluster-based rotation aims to identify a simple and well-clustered structure in a factor loading matrix (Yamamoto & Jennrich, 2013). The EFA was conducted because the current study involved a different target population—NGNs with 1 year of work experience from multiple European countries—compared with the original validation in 2003 among experienced nurses in Finland, and the factor structure could not be assumed to remain identical across these differing contexts. Cronbach’s alpha coefficient was used to determine the internal consistency of the total instrument and its categories. The small sample size did not allow country-by-country comparisons; therefore, the data are reported for the European cohort. Data were analyzed using the statistical program JASP (Version 0.19.3). A significance level of *p* < .05 was used in all statistical analyses.

## Ethical Considerations

The ethical approval for the COMPEUnurse research project was obtained from the Ethics Committee of the University of Turku, Finland (*Statement 16/2017, 6 March 2017*). Participants provided informed consent for the study by answering the informed consent item in the online questionnaire. The European Code of Conduct for Research Integrity (All European Academies, 2023) and the ethical principles of the Declaration of Helsinki (World Medical Association, 2024) were followed throughout the study.

## Results

A total of 239 NGNs from six European countries participated. Although sample sizes varied considerably between countries, the dataset is treated as a European sample for the purposes of this study. The mean age of NGNs was 27.9 years (*SD* = 7.6; range = 21–57). The majority of NGNs were female (85.8%). In total, 38.7% of NGNs had changed their workplace within the past 12 months, and 46.2% reported an intention to change their current workplace. Overall, 40.9% stated that they had never intended to leave the nursing profession. Most NGNs reported a moderate level of empowerment (57.5%), followed by those who reported a high level of empowerment (29.8%) (see Table 1).

**Table 1.** Sample Characteristics (*n* = 239).

Characteristics		*n*	%
Country	Czechia	22	9.2
	Finland	91	38.1
	Italy	39	16.3
	Portugal	32	13.4
	Slovakia	17	7.1
	Spain	38	15.9
Gender	Female	205	85.8
	Male	34	14.2
Intention to leave nursing profession	Never	97	40.9
	Fairly seldom	98	41.4
	Fairly often	31	13.1
	Very often	11	4.6
Intention to change the current workplace	Yes	110	46.2
	No	119	50.0
	Not working since graduation	9	3.8
Change working place within last 12 months	Yes	92	38.7
	No	136	57.1
	Not working since graduation	10	4.2
Satisfaction with the orientation in the current workplace	Fully agree	11	4.6
	Agree to some extent	75	31.7
	Neither agree nor disagree	91	38.4
	Disagree to some extent	42	17.7
	Fully disagree	5	2.1
	It does not concern me	13	5.5
Satisfaction with current job	Fully agree	1	0.4
	Agree to some extent	85	36.0
	Neither agree nor disagree	104	44.1
	Disagree to some extent	29	12.3
	Fully disagree	5	2.1
	It does not concern me	12	5.1
Satisfaction with quality of care in the current workplace	Fully agree	1	0.4
	Agree to some extent	78	33.1
	Neither agree nor disagree	107	45.3
	Disagree to some extent	31	13.1
	Fully disagree	5	2.1
	It does not concern me	14	5.9
Satisfaction with nursing as profession	Fully agree	104	43.9
	Agree to some extent	28	11.8
	Neither agree nor disagree	3	1.3
	Disagree to some extent	7	2.9
	Fully disagree	85	35.9
	Not working since graduation	10	4.2
Level of empowerment	Low level (<50)	30	12.8
	Moderate level (>50–75)	135	57.5
	High level (>75–100)	70	29.8

*Note.* Empowerment assessed with the VAS (Visual Analogue Scale) 0-100: 0–50 low empowerment; VAS >50–75 moderate; >75–100 high empowerment.

An attrition analysis was carried out to examine whether participants in this cross-sectional study differed from the original cohort of 1,742 respondents who participated a year earlier, indicating that younger individuals were less likely to take part in the current study (*p* < .0001).

### Level of Empowerment

The NGNs’ self-assessed level of empowerment was moderate, (67.2 out of 100, *SD* = 14.4). The highest level of empowerment was observed in the category Personal Integrity (*M* = 73.6, *SD* = 18.9), which belongs to the Impede element. The lowest level was in Sociability, part of the Performance element (*M* = 54.4, *SD* = 20.9). At the item level, the highest-rated items were Delegated responsibilities (*M* = 79.3, *SD* = 21.6), Confidence (*M* = 78.3, *SD* = 20.7), and Co-operation (*M* = 78.3, *SD* = 21.2), all of which correspond to a high level of empowerment (VAS >75–100). The lowest-rated item was Works for the common goal (*M* = 28.8, *SD* = 29.7), indicating a low level of empowerment (VAS 0–50) (see Table 2).

**Table 2.** NGNs’ Level of Empowerment.

Items	*n*	*Missing values*	*M*	*SD*
ELEMENT QUALITIES Category: Expertise			70.0	15.1
1. Autonomous	234	5	69.3	21.8
2. Has personal power	227	12	62.9	24.3
3. Competent 1 (in work on a general level)	234	5	73.5	19.8
4. Competent 2 (in daily nursing)	234	5	71.6	18.1
5. Personally responsible 1 (personal responsibility)	235	4	71.9	20.3
6. Personally responsible 2 (personal criticism)	234	5	71.1	22.8
Category: Future orientedness			65.3	20.0
7. Innovative, creative	230	9	58.2	24.8
8. Enthusiastic promoter	235	4	71.3	22.9
9. Forward thinking	230	9	67.1	25.1
ELEMENT: PERFORMANCE Category: Sociability			54.4	20.9
10. Discusses openly	232	7	70.0	24.0
11. Works for the common goal	125	114	28.8	29.7
12. Solves problems	217	22	51.3	27.4
ELEMENT: PROMOTE Category: Expertise			67.8	21.3
13. Evaluation and development	221	18	66.1	27.0
14. Co-operation	228	11	78.3	21.2
15. Training 1 (colleagues support in training)	212	27	63.5	27.5
16. Training 2 (opportunity for training)	215	24	66.8	29.0
ELEMENT: IMPEDE Category: Personal integrity			73.6	18.9
17. Delegated responsibilities	230	9	79.3	21.6
18. Confidence	231	8	78.3	20.7
19. Feedback	215	24	63.6	28.9
TOTAL			67.2	14.4

*Note.* Scale VAS 0 (*does not apply to me at all*) to 100 (*completely applies to me*).

Descriptions of the used item (1–19) are from Kuokkanen and Leino-Kilpi (2001).

### Association Between Empowerment and Background Factors

NGNs who expressed no intention to change their current workplace (*z*-value = −2.31, *p* = .02), or who had never considered leaving the nursing profession (χ^2^ = 19.65; *df* = 3; *p* < .01), reported significantly higher empowerment scores (total EENE score) than their counterparts. Statistically significant differences were also observed at the category level. NGNs with no intention to change their current workplace reported higher empowerment in Expertise (element Promote) and Personal Integrity (*z*-value = −2.15; *p* = .02 and *z*-value = −2.94; *p* < .01, respectively). In addition, those who had never intended to leave the nursing profession reported higher scores in Future Orientedness (χ^2^ = 26.86; *df* = 3; *p* < .01), Expertise (element Qualities; χ^2^ = 15.89; *df* = 3; *p* < .01), Expertise (element Promote; χ^2^ = 8.27; *df* = 3; *p* = .04), and Personal Integrity (χ^2^ = 13.53; *df* = 3; *p* < .01). Male NGNs assessed their empowerment in Expertise (element Qualities) significantly higher than female NGNs (*z*-value = −30.37, *p* < .01). The background factor Change of workplace within the last 12 months was not significantly associated with empowerment levels (see Table 3).

**Table 3.** Differences in the Total EENE Scores According to Background Factors.

Differences in total EENE					
	*n*	*M*	*SD*	*z-value*	*p*
Gender^a^					
Female	202	66.8	14.4	1.06	.29
Male	33	69.9	14.1		
Intention to change the current workplace^a^					
No	118	69.6	12.3	-2.31	.02*
Yes	108	64.8	15.8		
	*n*	*M*	*SD*	*Chi-squared χ* ^2^ *(df)*	*p*
Intention to leave nursing profession^b^					
Never	97	71.7	13.4	19.65 (3)	<.01*
Fairly seldom	30	59.2	14.7		
Fairly often	97	66.0	13.7		
Very often	10	60.8	15.4		
Change working place within last 12 months^b^					
No	135	67.1	14.2	1.57 (2)	.46
Not working since graduation	9	61.5	14.6		
Yes	90	68.0	14.8		
Differences in categories of EENE					
Gender^a^					
	*n*	*M*	*SD*	*z-value*	*p*
Expertise (element Qualities)					
Female	202	69.2	15.3	-30.37	<.01*
Male	33	74.9	13.5		
Future orientedness					
Female	202	64.7	19.9	1.06	.29
Male	33	68.9	20.0		
Sociability					
Female	202	53.9	20.5	0.37	.71
Male	33	57.1	23.7		
Expertise (element Promote)					
Female	200	73.4	18.7	0.63	.53
Male	33	75.0	20.1		
Personal integrity					
Female	200	67.9	21.3	-0.27	.78
Male	33	67.5	21.6		
Intention to change the current workplace^a^					
Expertise (element Qualities)					
No	118	71.0	14.8	-1.09	.27
Yes	108	69.0	15.2		
Future orientedness					
No	118	67.3	18.5	-1.53	.13
Yes	108	62.6	21.1		
Sociability					
No	118	55.9	18.6	-0.89	.38
Yes	108	53.1	23.1		
Expertise (element Promote)					
No	118	76.8	16.6	-2.15	.03*
Yes	107	70.7	20.6		
Personal integrity					
No	118	72.5	18.9	-2.94	<.01*
Yes	108	63.2	23.1		
Intention to leave nursing profession^b^					
	*n*	*M*	*SD*	*Chi-squared χ* ^2^ *(df)*	*p*
Expertise (element Qualities)					
Never	97	74.0	13.3	15.89 (3)	<.01*
Fairly seldom	30	61.6	15.1		
Fairly often	97	68.8	15.6		
Very often	10	65.9	17.3		
Future orientedness					
Never	97	71.6	17.6	26.86 (3)	<.01*
Fairly seldom	30	54.1	20.6		
Fairly often	97	64.2	19.5		
Very often	10	45.5	17.1		
Sociability					
Never	97	56.7	21.7	1.96 (3)	.58
Fairly seldom	30	50.1	22.8		
Fairly often	97	54.0	19.9		
Very often	10	50.7	16.3		
Expertise (element Promote)					
Never	97	77.1	17.8	8.27 (3)	.04*
Fairly seldom	30	67.9	19.1		
Fairly often	97	71.8	19.4		
Very often	10	75.3	19.4		
	*n*	*M*	*SD*	*Chi-squared χ* ^2^ *(df)*	*p*
Personal integrity					
Never	97	72.5	20.5	13.53 (3)	<.01*
Fairly seldom	30	57.5	21.2		
Fairly often	97	67.4	20.5		
Very often	9	59.8	25.0		
Change working place within last 12 months^b^					
Expertise (element Qualities)					
No	135	70.5	14.6	2.97 (2)	.23
Not working since graduation	9	61.6	14.6		
Yes	90	69.0	15.9		
Future orientedness					
No	135	64.4	19.8	0.59 (2)	.74
Not working since graduation	9	63.9	23.1		
Yes	90	66.5	20.1		
Sociability					
No	135	55.2	21.5	1.00 (2)	.61
Not working since graduation	9	49.0	18.0		
Yes	90	53.9	20.3		
Expertise (element Promote)					
No	134	72.3	18.8	3.09 (2)	.21
Not working since graduation	9	71.3	20.5		
Yes	90	75.9	18.9		
Personal integrity					
No	134	67.3	21.3	2.13 (2)	.34
Not working since graduation	9	61.6	18.0		
Yes	89	69.6	21.6		

a Mann–Whitney test. ^b^Kruskal–Wallis test.

* *p* < .05

At the category level, statistically significant positive correlations were found between Satisfaction with current job and Future Orientedness (*r* = .40; *p* < .01), Expertise (element Promote) (*r* = .31; *p* < .01), Expertise (element Qualities) (*r* = .29; *p* = .01), and Personal Integrity (*r* = .35; *p* < .01). Similarly, Satisfaction with orientation in the current workplace was positively correlated with Personal Integrity (*r* = .26; *p* < .01) and Expertise (element Promote) (*r* = .20; *p* = .04). Age was positively correlated with Expertise (element Qualities) (*r* = .15; *p* = .02), Future Orientedness (*r* = .20; *p* < .01), and Sociability (*r* = .17; *p* < .01) (see Table 4).

**Table 4.** Correlations of EENE Categories with NGNs’ Age and Satisfaction-Related Background Factors.

	EENE categories				
Background variables	Expertise (element Qualities)	Future orientedness	Sociability	Expertise (element Promote)	Personal integrity
Age^a^	0.15* *p* = .02	0.20* *p* < .01	0.17* *p* < .01	0.12 *p* = .05	0.02 *p* = .71
Satisfaction with the orientation in workplace^b^	0.18 *p* = .05	0.18 *p* = .06	0.12 *p* = .20	0.20* *p* = .04	0.26* *p* < .01
Satisfaction with current job^b^	0.29* *p* < .01	0.40* *p* < .01	0.17 *p* = .07	0.31* *p* < .01	0.35* *p* < .01
Satisfaction with quality of care^b^	-0.06 *p* = .50	0.03 *p* = .73	-0.02 *p* = .83	0.07 *p* = .46	0.10 *p* = .31
Satisfaction with nursing as profession^b^	0.11 *p* = .26	0.16 *p* = .09	-0.02 *p* = .82	0.15 *p* = .12	0.12 *p* = .20

a Spearman correlation coefficient. ^b^Pearson correlation coefficient.

* *p* < .05.

### Validity

#### EFA of EENE

The Kaiser–Meyer–Olkin measure was adequate (0.89), exceeding the recommended minimum threshold of 0.60. Bartlett’s test of sphericity (χ^2^ = 1932.43; *df* = 171; *p* < .01) confirmed the suitability of the data for factor analysis. The goodness-of-fit measure was χ^2^ = 383.05; *df* = 129; *p* < .01. The EFA revealed four factors with eigenvalues greater than 1 (6.95; 1.85; 1.32; 1.16). Cluster rotation was applied. The total variance explained was approximately 59%, with the first factor explaining 19.9%, the second 17.2%, the third 14.2%, and the fourth 8.2% of the variance. Factor 1 comprised six items, with the highest loading observed for item 14 (Co-operation) (loading = 0.96). Factor 2 also included six items, Factor 3 comprised four items, and Factor 4 included two items. Although the number of factors matched those identified in previous analyses, not all items loaded onto the same factors as in the original instrument (Table 5).

**Table 5.** Exploratory Factor Analysis.

Items	Factor 1	Factor 2	Factor 3	Factor 4	Uniqueness
14. Co-operation	0.962				0.208
17. Delegated responsibilities	0.805				0.431
13. Evaluation and development	0.653				0.419
8. Enthusiastic promoter	0.504				0.524
10. Discusses openly	0.463				0.547
18. Confidence	0.448				0.557
3. Competent 1		0.891			0.390
1. Autonomous		0.796			0.511
5. Personally responsible 1		0.514			0.450
4. Competent 2		0.514			0.548
6. Personally responsible 2		0.408			0.746
2. Has personal power		0.402			0.599
7. Innovative, creative			0.675		0.434
12. Solves problems			0.634		0.483
9. Forward thinking			0.566		0.421
11. Works for the common goal			0.483		0.823
15. Training 1				0.814	0.288
16. Training 2				0.536	0.565
19. Feedback	-	-	-	-	-

*Note.* Applied rotation method is cluster–oblique rotation (Yamamoto & Jennrich, 2013).

Descriptions of the used item (1–19) are from Kuokkanen and Leino-Kilpi (2001).

#### Construct Validity

To support construct validity, inter-category correlations within the EENE were examined. All correlations were statistically significant, with the strongest observed between Expertise (element Qualities) and Future Orientedness (*r* = 0.63; *p* < .01) and between Expertise (element Promote) and Personal Integrity (*r* = .62; *p* < .01). These findings suggest that theoretically related constructs are moderately associated, supporting the EENE instrument’s robust psychometric properties for assessing empowerment among NGNs in an international context.

### Internal Consistency

Cronbach’s alpha values for the individual categories ranged from 0.62 (*Sociability*) to 0.84 (*Personal Integrity*). The overall Cronbach’s alpha for the instrument was 0.88, indicating acceptable internal consistency for use with new instruments (DeVon et al., 2007).

## Discussion

### Level of Empowerment

This study aimed to describe the level of empowerment among NGNs 1 year post-graduation across six European countries and to evaluate the psychometric properties of the EENE instrument within this early-career, international cohort. Most NGNs reported moderate empowerment, consistent with findings by Sarıköse and Çelik (2024), who observed moderate psychological and structural empowerment among NGNs with less than 12 months of experience. In our sample, NGNs who expressed greater enthusiasm for their work and reported having more creative ideas applicable to their practice (category: Future Orientedness) were more satisfied with their current workplace. These findings are encouraging, as higher empowerment is associated with improved competence, confidence, and job satisfaction (Masias et al., 2020).

Although NGNs’ assessments varied across categories, empowerment remained consistently moderate. The highest assessments were observed in Personal integrity (delegated responsibilities, confidence, and feedback), potentially reflecting effective organizational support, orientation, and supervision (Baharum et al., 2023). Fewer than one in five NGNs expressed dissatisfaction with their orientation, while the remainder were either satisfied or neutral. Previous research has shown that satisfaction with orientation programs correlates with higher structural empowerment (Sarıköse & Çelik, 2024). However, as organizational support and supervisory practices were not directly examined in this study, this interpretation remains speculative and warrants further investigation.

The lowest assessments were in Sociability, defined as a nurse who “discusses openly, works for the common goal, and solves problems” (Kuokkanen, 2003, p. 42). This mirrors findings from our earlier study at graduation (Visiers-Jiménez et al., 2022). It may reflect a sense of hesitation among NGNs to express opinions or take initiative in collaborative problem-solving situations, due to a lack of confidence or fear of making mistakes. In hierarchical health care environments, unclear expectations and lack of confidence may inhibit open dialogue (Salas et al., 2018). These dynamics may be reinforced by broader cultural norms around authority, teamwork, and communication—particularly those described in Hofstede’s 6-D Model of National Culture (2010). In high power distance cultures such as Slovakia, Portugal, Spain, the Czech Republic, and Italy, hierarchy is accepted and respected, which may constrain participative collaboration. In contrast, Finland’s more decentralized structures encourage consultation and direct communication. Furthermore, the individualist orientation common to all participating countries emphasizes personal responsibility and achievement over collective motivation, which may reduce engagement in common goals (Hofstede et al., 2010). Without structured mentorship and team-based learning, NGNs may therefore struggle to develop the communication and collaboration skills needed for effective teamwork (Baharum et al., 2023). Nurse managers play a crucial role in fostering open communication and should actively involve NGNs in team discussions to support the development of reflective dialogue and professional confidence.

We also identified significant associations between empowerment and some factors. NGNs with no intention to change their current workplace reported significantly higher empowerment than those considering a change, aligning with previous findings (Masias et al., 2020). Intentions to leave the nursing profession may reflect perceived lack of control, limited development opportunities, or dissatisfaction with the work environment (Ding & Wu, 2023; Lyu et al., 2018). Addressing career concerns and fostering growth opportunities can enhance retention and job satisfaction (Gong et al., 2022; Perkins et al., 2023). Empowerment is linked to positive practice environments, including organizational justice, leadership support, and professional development (Kuokkanen et al., 2014). Lower empowerment among those intending to leave may indicate deficiencies in these areas. Empowered nurses contribute positively to patient safety, satisfaction, and organizational performance (Ferreira Aydogdu, 2024; Goedhart et al., 2017). Our result highlights the relevance of early-career support and empowering leadership in retaining NGNs and fostering stability in the workforce.

NGNs who had never considered leaving the nursing profession reported higher empowerment, consistent with earlier studies (Favaro et al., 2021; Kuokkanen et al., 2016). This was reflected in higher scores in Future Orientedness, which was positively associated with satisfaction with current job and may indicate a long-term commitment to nursing. Similarly, higher assessments in Expertise (element Promote) and Personal integrity were associated with both satisfaction with current job and satisfaction with orientation in current workplace, suggesting that supportive environments and opportunities for development reinforce professional commitment (Hjazeen et al., 2024; Yesilbas & Kantek, 2024). Moreover, male NGNs reported higher empowerment in Expertise (element Qualities), which may reflect culturally shaped gender role expectations in European nursing. Men may experience greater pressure to demonstrate competence or may be perceived as more authoritative within traditionally female-dominated contexts (Romem & Rozani, 2024).

From a transcultural nursing perspective, these findings also align with Leininger’s Culture Care Diversity and Universality theory (Leininger, 2001; Leininger & McFarland, 2006), which emphasizes that culturally congruent care arises when nurses understand and act upon patients’ cultural values, beliefs, and life contexts. Empowerment can thus be viewed as a prerequisite for culturally responsive practice: empowered nurses are more confident to engage in open dialogue, adapt care to diverse needs, and advocate for equitable treatment. In multicultural European health care settings, strengthening NGNs’ empowerment may not only enhance satisfaction and retention but also support the delivery of person-centered, culturally congruent care across different cultural and organizational contexts.

### Validity

The psychometric evaluation of the EENE instrument in this international sample supports its construct validity and factorial structure (Kuokkanen, 2003). With 239 participants and 19 items, the sample meets accepted criteria for EFA, which recommends 5–10 participants per item (Costello & Osborne, 2005). The Kaiser–Meyer–Olkin value exceeded the recommended threshold (Kaiser, 1974), and Bartlett’s test of sphericity confirmed the sufficiency of inter-item correlations. EFA revealed a four-factor structure with eigenvalues greater than one, explaining approximately 59% of the total variance—an acceptable level (Watkins, 2018). Factor loadings were strong, and cluster rotation clarified the structure, although some items loaded differently from those in the original instrument (Kuokkanen, 2003). This may be due to differences in population and context: the original study involved experienced Finnish nurses, whereas the present sample comprised NGNs from multiple European countries. Such differences in item loading may reflect not only variations in career stage but also the influence of cultural and health care system characteristics across countries.

Cultural, linguistic, and experiential differences, as well as changes in nursing roles over two decades, may have influenced item interpretation. One item (Feedback) did not load substantially onto any factor, suggesting it may not resonate with NGNs in an international context and highlighting the need for further cross-cultural validation. It is possible that the concept of “feedback” is interpreted differently across nursing cultures—ranging from formal and expected to informal or absent—which could influence how NGNs responded to the item and potentially affected its construct validity. Future adaptations may need to reword or recontextualize the item to ensure cultural relevance and conceptual clarity across settings.

Construct validity was further supported by statistically significant inter-category correlations, with theoretically related categories showing moderate associations (Schober et al., 2018). Overall, the EENE instrument demonstrated a coherent factor structure and acceptable internal consistency (DeVon et al., 2007), confirming its reliability for assessing empowerment among NGNs in diverse international settings.

### Strengths and Limitations

This is the first study to apply the EENE instrument to NGNs 1 year post-graduation and to evaluate its psychometric properties within a multinational European context. However, this study has limitations. As part of a larger longitudinal research project, the sample size diminished over time due to participant attrition, making country-specific analyses unfeasible. Consequently, the findings are presented as a European-level cohort, and the individual contribution of each country to the results remains unknown. Although the study encompasses six countries, treating the cohort as a single European sample may obscure important cultural variations in empowerment. Data collection continued until the end of November 2020, coinciding with the first wave of the COVID-19 pandemic in Europe. This period was marked by significant disruptions to health care systems and increased demands on professionals. It has also been reported that the youngest nurses were among those most affected by the COVID-19 pandemic (Sherman, 2022). This context may also help explain the findings from the attrition analysis, which indicated that the youngest respondents were less likely to participate in the present study, even though they had been part of the larger cohort 1 year earlier. Consequently, the pandemic may have influenced NGNs’ capacity or willingness to participate, as well as key variables such as empowerment, orientation, and job satisfaction. The COVID-19 context should therefore be acknowledged as a limitation when interpreting the findings. In addition, the use of convenience sampling limits the generalizability of the findings, and only preliminary conclusions can be drawn. Despite these limitations, the study provides valuable insights into NGNs’ empowerment and informs future multicountry research.

## Conclusion

NGNs from six European countries report a moderate level of empowerment after their initial year in clinical practice. Empowerment is positively associated with job satisfaction and intentions to remain in both the profession and the workplace, underscoring its significance for early-career workforce retention. NGNs felt most empowered in areas related to Expertise, while Sociability emerged as the lowest-rated domain, indicating a need for enhanced support in team communication and collaboration. These findings highlight the importance of structured orientation programs that respect local cultural norms, promote empowering leadership, and support inclusive work environments. Such programs play a key role in fostering the development of NGNs, including the need to tailor empowerment strategies to cultural expectations related to autonomy, teamwork, and leadership. The EENE instrument demonstrated robust psychometric properties in this international sample. The four-factor structure, acceptable explained variance, and strong factor loadings support its construct validity and internal consistency. These findings support its suitability for assessing NGNs’ empowerment and highlight its potential as a tool for cross-cultural workforce assessment across diverse European settings.
